# Prediction and early biomarkers of cognitive decline in Parkinson disease and atypical parkinsonism: a population-based study

**DOI:** 10.1093/braincomms/fcac040

**Published:** 2022-03-15

**Authors:** David Bäckström, Gabriel Granåsen, Susanna Jakobson Mo, Katrine Riklund, Miles Trupp, Henrik Zetterberg, Kaj Blennow, Lars Forsgren, Magdalena Eriksson Domellöf

**Affiliations:** 1 Department of Clinical Science, Neurosciences, Umeå University, Umeå, Sweden; 2 Department of Neurology, Yale University, New Haven, CT, USA; 3 Epidemiology and Global Health Unit, Department of Public Health and Clinical Medicine, Umeå University, Umeå, Sweden; 4 Department of Radiation Sciences, Diagnostic Radiology and Umeå Center for Functional Brain Imaging, Umeå University, Umeå, Sweden; 5 Department of Psychiatry and Neurochemistry, Institute of Neuroscience and Physiology, The Sahlgrenska Academy at the University of Gothenburg, Gothenburg, Sweden; 6 Clinical Neurochemistry Laboratory, Sahlgrenska University Hospital, Mölndal, Sweden; 7 Department of Neurodegenerative Disease and UCL Queen Square Institute of Neurology, London, UK; 8 UK Dementia Research Institute at UCL, London, UK; 9 Department of Psychology, Umeå University, Umeå, Sweden

**Keywords:** cognitive decline, dementia, Parkinson disease, multiple system atrophy, progressive supranuclear palsy

## Abstract

The progression of cognitive decline is heterogeneous in the three most common idiopathic parkinsonian diseases: Parkinson disease, multiple system atrophy and progressive supranuclear palsy. The causes for this heterogeneity are not fully understood, and there are no validated biomarkers that can accurately identify patients who will develop dementia and when. In this population-based, prospective study, comprehensive neuropsychological testing was performed repeatedly in new-onset, idiopathic parkinsonism. Dementia was diagnosed until 10 years and participants (*N* = 210) were deeply phenotyped by multimodal clinical, biochemical, genetic and brain imaging measures. At baseline, before the start of dopaminergic treatment, mild cognitive impairment was prevalent in 43.4% of the patients with Parkinson disease, 23.1% of the patients with multiple system atrophy and 77.8% of the patients with progressive supranuclear palsy. Longitudinally, all three diseases had a higher incidence of cognitive decline compared with healthy controls, but the types and severity of cognitive dysfunctions differed. In Parkinson disease, psychomotor speed and attention showed signs of improvement after dopaminergic treatment, while no such improvement was seen in other diseases. The 10-year cumulative probability of dementia was 54% in Parkinson disease and 71% in progressive supranuclear palsy, while there were no cases of dementia in multiple system atrophy. An easy-to-use, multivariable model that predicts the risk of dementia in Parkinson disease within 10 years with high accuracy (area under the curve: 0.86, *P* < 0.001) was developed. The optimized model adds CSF biomarkers to four easily measurable clinical features at baseline (mild cognitive impairment, olfactory function, motor disease severity and age). The model demonstrates a highly variable but predictable risk of dementia in Parkinson disease, e.g. a 9% risk within 10 years in a patient with normal cognition and CSF amyloid-β_42_ in the highest tertile, compared with an 85% risk in a patient with mild cognitive impairment and CSF amyloid-β_42_ in the lowest tertile. Only small or no associations with cognitive decline were found for factors that could be easily modifiable (such as thyroid dysfunction). Risk factors for cognitive decline in multiple system atrophy and progressive supranuclear palsy included signs of systemic inflammation and eye movement abnormalities. The predictive model has high accuracy in Parkinson disease and might be used for the selection of patients into clinical trials or as an aid to improve the prevention of dementia.

## Teaser

Bäckström *et al.* report that, in a population-based cohort of patients with new-onset Parkinson disease, approximately half develop dementia within 10 years. Measurement of CSF biomarkers together with baseline cognitive function, olfaction and motor disease severity has high accuracy for predicting who will develop dementia.

## Graphical Abstract

**Graphical Abstract:**
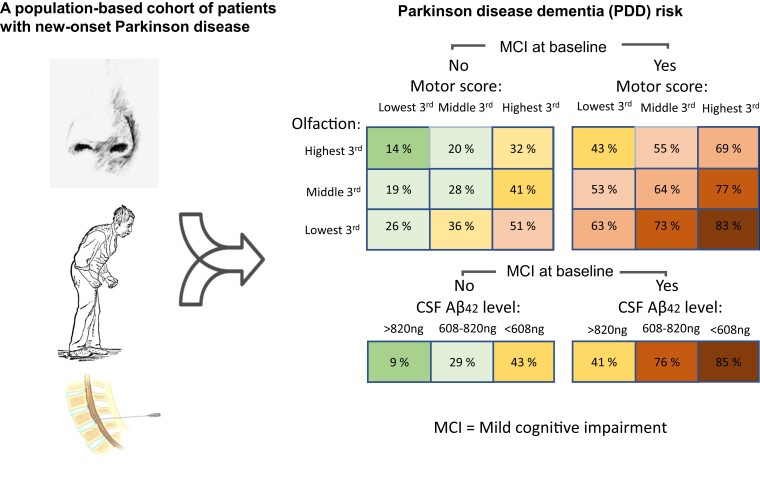

## Introduction

Dementia is one of the most devastating non-motor features of Parkinson disease and progressive supranuclear palsy (PSP) but appears to be less common in multiple system atrophy (MSA).^1,2^ When occurring, it is associated with an impaired quality of life, increased morbidity and a shortened lifespan.^3^

In Parkinson disease, studies have shown that dementia develops in between 46% and at least 80% of the patients over the course of the disease, depending on study design, but with high variability in severity and time of onset.^4,5^ A variety of mechanisms are proposed to contribute to cognitive decline and dementia in Parkinson disease, including lack of dopamine and acetylcholine, aggregation of misfolded protein species, in particular α-synuclein and β-amyloid in limbic and cortical networks, synaptic dysfunction, axonal degeneration, neuroinflammation, brain atrophy and disturbance of brain connectivity.^2,6–8^ Furthermore, recent evidence shows that distinct strains of pathological α-synuclein or genetic haplotypes may explain some of the variability in the cognitive phenotype.^9,10^ Some ‘seed’ strains of α-synuclein may have a propensity for wide-spread distribution in cortical networks, which could explain why some patients with Parkinson disease (and dementia with Lewy bodies) are affected by early dementia. Robust evidence also shows that co-incident β-amyloid deposition in the brain affects cognitive function negatively in Parkinson disease, is associated with Parkinson disease dementia (PDD), and that this pathology may promote the spread of α-synuclein.^6,11,12^ This can further contribute to the heterogeneity of cognitive dysfunction.

Clinical factors associated with PDD have been identified, with consistent associations found for preexisting mild cognitive impairment (MCI), hyposmia, hallucinations, high overall severity of motor symptoms, speech impairment, older age at onset, axial impairment and gait difficulty^13–15^ and biomarker patterns such as low Aβ42 in the CSF.^16–18^ Prognostic models incorporating a few or several of these factors have been developed^18–20^ but have not been validated for patients followed for longer than 2–5 years, during periods when most Parkinson disease patients will develop PDD. Furthermore, there are presently no validated biomarker patterns that can accurately identify patients who will develop dementia and when. Given the phenotypic variability of Parkinson disease, and the likely need to initiate disease-modifying treatments in the early phase, reliable diagnostic and prognostic biomarkers are urgently needed. Aerobic exercise seems to have a small effect in preventing cognitive decline.^21,22^ Beyond this, the effects of risk factors for PDD that are potentially preventable have not been thoroughly investigated.

While both Parkinson disease and MSA are α-synucleinopathies, PSP is neuropathologically characterized by intracellular, hyper-phosphorylated, four-repeat tau inclusions in neurons and glia.^23^ The tau pathology is mainly affecting midbrain and deep brain nuclei in the early course of the disease and spreading to cortical regions during disease progression.^24^ Cognitive dysfunction and dementia are core features of PSP, with cognitive dysfunction affecting 40–62% of patients in prospective studies,^1^ and is linked with increasing cortical tau burden, cholinergic deficiency and neuroinflammation.^25^

**Figure 1:**
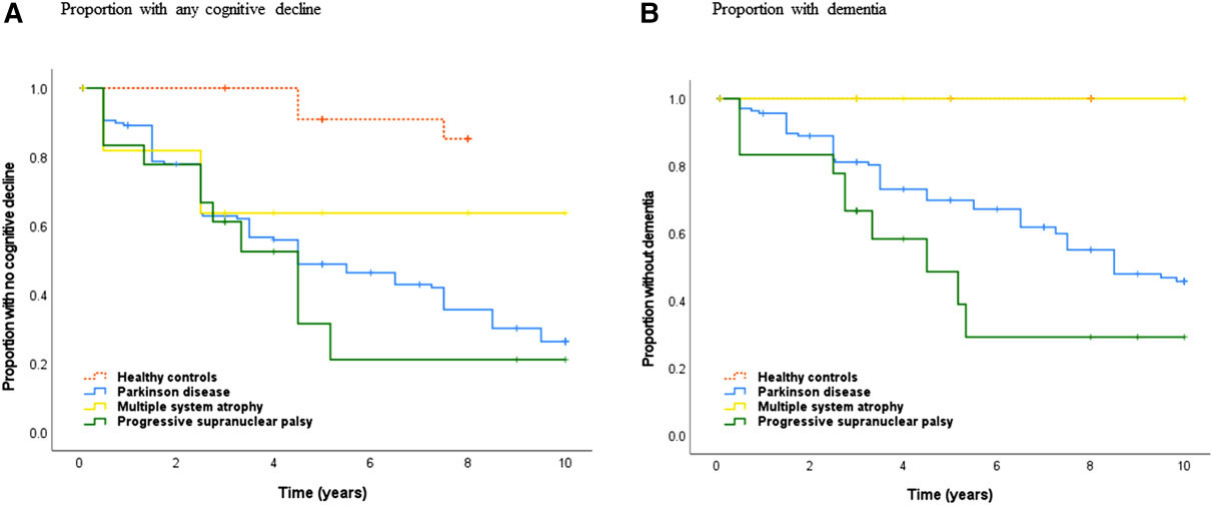
**Kaplan–Meier graphs showing the incidence of cognitive decline and dementia.** Time is measured in years from first visit (baseline). No participant had dementia at baseline. The Kaplan–Meier graphs show incident (**A**) cognitive decline (MCI or dementia) and (**B**) dementia within 10 years. There were significant between-groups differences for (**A**) cognitive decline (*P* < 0.001, log rank) and (**B**) dementia within 10 years (*P* < 0.001, log rank). For specific group comparisons, see the ‘Results’ section.

**Figure 2:**
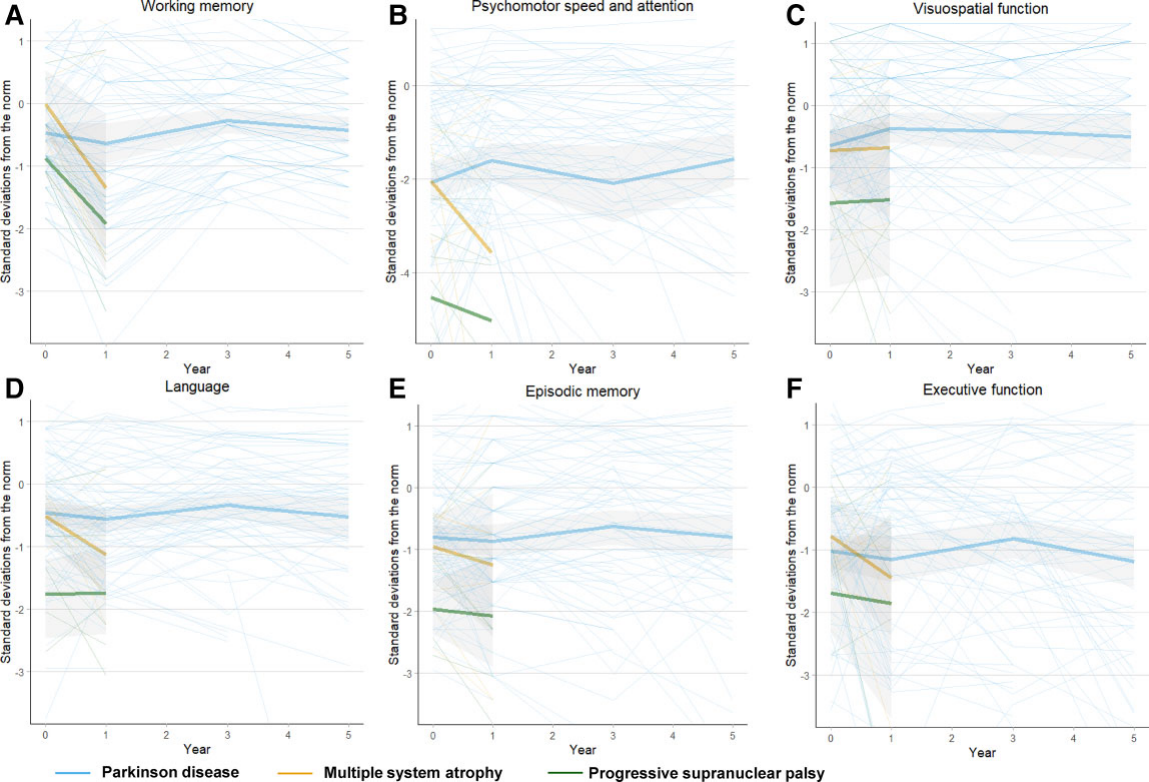
**Dysfunction in specific cognitive domains in idiopathic parkinsonism.** Cognitive test results in each cognitive domain (**A**, working memory; **B**, psychomotor speed and attention; **C**, visuospatial function; **D**, language; **E**, episodic memory; **F**, executive function) measured in SDs from the scores of the HCs (norm) at baseline. Test results are shown both for individuals (thin lines) and for diagnostic group means (thick lines), as the mean is affected by selection bias over time. Difference between HCs and (i) Parkinson disease (for **A**, mean SD: −0.49, *P* = 0.002; **B**, mean SD: −2.19, *P* < 0.001; **C**, mean SD: −0.67, *P* = 0.003; **D**, mean SD: −0.50, *P* = 0.004; **E**, mean SD: −0.87, *P* < 0.001; **F**, mean SD: −1.13, *P* < 0.001), (ii) MSA (for **A**, mean SD: −0.30, n.s.; **B**, mean SD: −2.56, *P* < 0.001; **C**, mean SD: −0.46, n.s.; **D**, mean SD: −0.50, n.s.; **E**, mean SD: −0.84, *P* = 0.033; **F**, mean SD: −0.98, *P* = 0.003) and (iii) PSP disease (for **A**, mean SD: −1.15, *P* = 0.002; **B**, mean SD: −4.44, *P* < 0.001; **C**, mean SD: −1.74, *P* < 0.001; **D**, mean SD: −1.65, *P* < 0.001; **E**, mean SD: −1.85, *P* < 0.001; **F**, mean SD: −1.56, *P* < 0.001) is between-groups mean differences estimated by linear mixed models. In the figure, the mean is only shown until 1 year for MSA and PSP because of few datapoints beyond this time point. For tests included in each domain, see Supplementary material. See also Table 2. Grey area, 95% CI; Year 0, baseline; n.s., non-significant.

**Figure 3:**
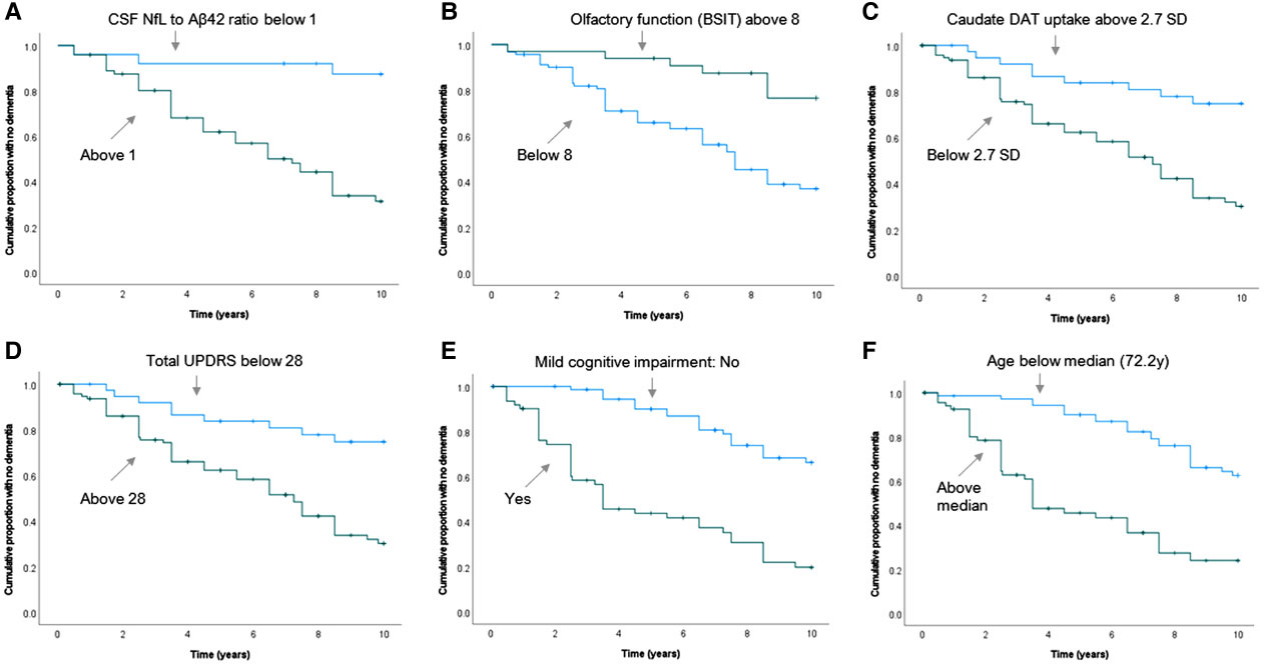
**Baseline predictors of dementia in Parkinson disease within 10 years.** Six early predictors (**A–F**) of PDD. The Kaplan–Meier graphs show the effect of baseline predictors above and below specified cut-offs on PDD risk. All cut-offs were made at the highest Youden Index. NfL, neurofilament light chain protein; Aβ42, amyloid-β 42; DAT, dopamine transporter; UPDRS, Unified Parkinson’s Disease Rating Scale. *P*-value < 0.001 (log rank) for all predictors, **A** through **F**).

**Figure 4:**
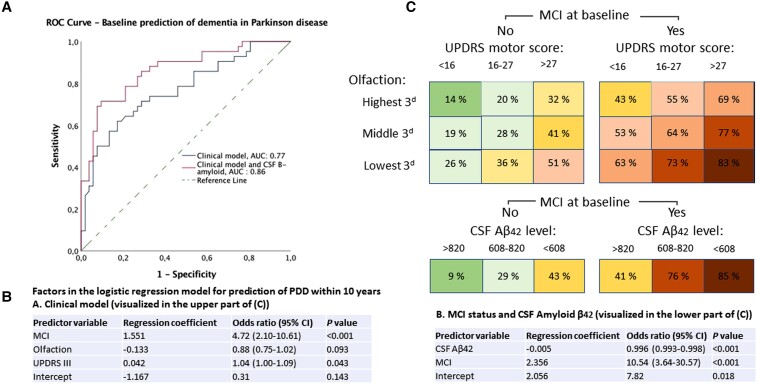
**Prediction of dementia in Parkinson disease within 10 years, by logistic regression.** (**A**) Receiver operating characteristic curves with AUC for predicting in Parkinson disease (PDD) with the clinical model and the combined clinical-biomarker model. Asymptotic significance for having a better predictive capability than chance, *P* < 0.001 (both models). (**B**) List of factors (using three factors versus two factors, to the left and right, respectively) in the logistic regression model for prediction of dementia. (**C**) A risk chart with the individual risk of dementia within 10 years, shown for a patient in the middle value of each tertile of the risk factors shown in the predictive models in **B**. MCI, mild cognitive impairment; UPDRS, Unified Parkinson’s Disease Ranking Scale (part III was the motor subscale used for motor scores); CSF Aβ42, amyloid-β42 in CSF.

The evolution of cognitive dysfunction in the idiopathic atypical parkinsonian diseases, defined here as MSA and PSP, is less well studied compared with Parkinson disease, especially in population-based settings. Knowing the different trajectories for cognitive dysfunction in all three diseases is critical for (i) counselling individual patients and (ii) measuring the right outcomes in clinical trials. In this prospective study, cognitive impairments in the three most common idiopathic parkinsonian disorders were comprehensively assessed, throughout 10 years, in deeply phenotyped patients recruited through a population-based screening procedure. The study is an effort to further stratify parkinsonian patients based on clinical and molecular biomarkers to predict cognitive impairments (see Tables 1–4 and Figs 1–4).

**Table 1:** Demographic and clinical characteristics of the study cohort at baseline

Variable	HC (*n* = 36)		PD (*n* = 143)		MSA (*n* = 13)		PSP (*n* = 18)		*P*-value
	Mean	(SD)	Mean	(SD)	Mean	(SD)	Mean	(SD)	
Age (years)	68.2	(6.6)	71.2	(9.9)	73.6	(9.6)	75.0	(7.1)	0.020
Sex (male/female)	20/16		85/58		8/5		8/10		0.002
Education (years)	11.6	(3.5)	10.0	(4.1)	8.7	(3.6)	8.2	(3.1)	0.009
Duration (years)			1.8	(1.4)	1.9	(0.9)	2.1	(1.5)	0.507
MMSE	29.1	(0.8)	28.5	(1.7)	29.0	(1.1)	27.9	(1.6)	0.104
MADRS	0.3	(0.5)	4.5	(3.7)	8.0	(7.4)	7.7	(9.4)	<0.001
UPDRS III			27.6	(11.6)	27.8	(12.1)	29.3	(11.8)	<0.001
UPDRS Tot			38.1	(14.6)	39.5	(16.9)	39.2	(14.7)	0.947
H&Y stage			2.3	(0.7)	2.7	(0.8)	2.8	(0.9)	0.004
Olfactory function	9.5	(2.0)	6.5	(2.7)	7.0	(3.3)	7.1	(2.3)	<0.001
Syst. blood pressure			146	(20)	149	(22)	150	(23)	0.250
Syst. pressure drop			11	(19)	21	(22)	2	(15)	0.025
Ever smoker, *n* (%)			48 (34.0%)		7 (53.8%)		3 (18.8%)		0.145
MCI, *n* (%)	4 (11.8%)		62 (43.4%)		3 (23.1%)		14 (77.8%)		<0.001
Alcohol use, *n* (yes/no)			82/60		9/4		7/9		0.417
*Biomarkers*									
BMI	25.6	(4.7)	26.0	(3.6)	25.0	(3.0)	28.9	(3.9)	0.250
*APOE* ɛ4 carrier	10/21		31/100		0/13		4/14		0.299
CSF NfL	895	(283)	1446	(1216)	2028	(1740)	2562	(1516)	<0.001
CSF Aβ_42_	826	(230)	715	(225)	639	(261)	565	(193)	0.003
CSF NFL to Aβ_42_ ratio	1.3	(0.8)	2.3	(2.0)	3.8	(3.3)	4.7	(2.4)	<0.001
CSF α-synuclein	0.8	(0.3)	0.8	(0.4)	0.9	(0.4)	0.6	(0.3)	0.230
CSF tau	304	(139)	299	(189)	270	(118)	257	(105)	0.745
Blood leucocytes	—	—	6.4	(1.5)	5.5	(1.7)	6.9	(1.7)	0.062
Blood TSH	—	—	1.88	(1.38)	1.81	(1.01)	2.38	(3.19)	0.942
Blood B12	—	—	420	(230)	597	(486)	506	(258)	
Blood folate	—	—	21.4	(12.9)	21.1	(12.1)	23.0	(13.9)	0.900
DAT deficit, caudate	0.0	(1.0)	−2.7	(1.1)	−2.8	(1.5)	−3.3	(1.0)	<0.001
DAT deficit, putamen	0.0	(1.0)	−3.9	(0.9)	−3.7	(.9)	−4.2	(0.9)	<0.001

Data are means and SDs from 36 HCs and 174 patients with new-onset idiopathic Parkinson disease, MSA and PSP. Olfactory function was measured by the number of correct answers on the 12-item brief smell inventory test. *P*-values for group differences are calculated by the Kruskal–Wallis test (non-normal distributions), ANOVA (normal distributions) or *χ*^2^. MMSE, mini-mental state examination; MADRS, Montgomery–Åsberg depression scale; UPDRS, Unified Parkinson disease rating scale; H&Y stage, Hoehn and Yahr stage; MCI, mild cognitive impairment; NfL, neurofilament light chain protein; Aβ142, β-amyloid 42; DAT, dopamine active transporter.

**Table 2:** Dysfunctions in specific cognitive domains in idiopathic parkinsonism

	PD			MSA			PSP		
	Mean	SD	*P*-value (PD versus HC)	Mean	SD	*P*-value (MSA versus PD)	Mean	SD	*P*-value (PSP versus PD)
*(A) Baseline differences*									
Working memory	−0.47	0.85	0.017*	−0.01	0.92	0.103	−0.88	0.67	0.052
Psychomotor speed and attention	−2.08	2.25	<0.001*	−2.04	2.45	0.816	−4.52	3.57	0.003*
Visuospatial function	−0.65	1.31	0.008*	−0.72	0.97	0.696	−1.58	2.36	0.189
Language	−0.47	0.93	0.005*	−0.52	0.69	0.979	−1.76	1.32	<0.001*
Episodic memory	−0.81	1.04	<0.001*	−0.95	0.97	0.641	−1.96	0.89	<0.001*
Executive function	−0.97	1.35	<0.001*	−0.67	1.01	0.725	−1.52	1.19	0.072
	**Diff.**	***P-*value (PD versus HC)**		**Diff.**	***P-*value (MSA versus HC)**		**Diff.**	***P-*value (PSP versus HC)**	
*(B) Longitudinal differences (estimated by mixed models)*									
Working memory	−0.49	0.002*		−0.30	0.632		−1.15	0.002*	
Psychomotor speed and attention	−2.19	<0.001*		−2.56	<0.001*		−4.44	<0.001*	
Visuospatial function	−0.67	0.003*		−0.46	0.257		−1.74	<0.001*	
Language	−0.50	0.004*		−0.50	0.068		−1.65	<0.001*	
Episodic memory	−0.87	<0.001*		−0.84	0.033*		−1.85	<0.001*	
Executive function	−1.13	<0.001*		−0.98	0.003*		−1.56	<0.001*	

The performance in cognitive domains is showed in *z*-scores, where the mean of all test results in a domain is in SDs from the mean of the HCs at baseline. At the baseline cross-sectional comparison, Parkinson disease was compared with the HCs, while PSP and MSA were only compared with Parkinson disease, as this was determined to be clinically most relevant. The difference in table section (B) (Diff.) is estimated marginal means, given as the total difference from HCs throughout all follow-ups, after adjustment for age and sex. Mixed models were calculated with intercept as a random effect, autoregressive 1.

* The variable was significant after controlling for false discovery rate with *α* = 0.05, by the Benjamini–Hochberg procedure. TMT A is the measure for psychomotor speed and attention.

**Table 3:** Predictors of dementia in Parkinson’s disease within 10 years

	Clinical model	Univariate		Multivariable	
Phenotype, at baseline	Observed range	HR (95% CI)	*P*-value	HR (95% CI)	*P*-value
Age	39.7–90.0 years	1.07 (1.04–1.10)	<0.001*	1.05 (1.01–1.09)	0.014
Years of education	6–30 years	0.9 (0.8–1.0)	0.019*		
Smoker	Ever versus never	0.9 (0.5–1.6)	0.740		
MADRS score	0–18	1.0 (1.0–1.1)	0.147		
Sex	Male versus female	1.1 (0.6–1.7)	0.820		
H&Y stage	1.0–5.0	2.0 (1.4–2.8)	<0.001*		
UPDRS total score	8–81	1.0 (1.0–1.1)	<0.001*		
UPDRS III subscore	5–62	1.0 (1.0–1.1)	<0.001*	1.0 (1.0–1.1)	0.004
PIGD score	0–2.4	3.2 (1.8–5.7)	<0.001*		
Tremor score	0–1.5	0.6 (0.3–1.3)	0.220		
Blink frequency	0–80 per minute	1.0 (1.0–1.0)	0.029*		
Slow saccades	Yes versus no	1.2 (0.6–2.3)	0.463		
Postural instability first year	Yes versus no	4.6 (2.2–9.7)	<0.001*		
Symmetrical onset	Yes versus no	2.3 (2.2–3.9)	0.003*		
MCI	Yes versus no	4.5 (2.7–7.6)	<0.001*	4.2 (2.2–7.7)	<0.001
Olfactory function	0–12 correct	0.8 (0.7–0.9)	<0.001*	0.8 (0.7–0.9)	<0.001
Alcohol use	Yes versus no	0.5 (0.3–0.9)	0.014*		
Orthostatic blood pressure drop	−22 to 107 mmHg	1.0 (1.0–1.0)	0.006*		
**Biomarkers, at baseline**	**Biomarker model**				
BMI	19.2–36.5	1.1 (1.0–1.2)	0.035		
*APOE* ɛ4 genotype	0–2 ɛ4 alleles	1.0 (0.6–1.6)	0.851		
*MAPT* haplotype	H1/H2	1.0 (0.6–1.8)	0.923		
CSF NfL (pg/ml)	271–8700	1.0 (1.0–1.0)	<0.001*		
CSF Aβ_42_ (pg/ml)	249–1373	1.0 (1.0–1.0)	<0.001*		
CSF NfL to Aβ_42_ ratio	0.4–10.7	1.3 (1.2–1.7)	<0.001*	1.5 (1.2–1.7)	<0.001
CSF α-synuclein (pg/ml)	0.3–2.2	1.1 (0–1.0)	0.762		
B-TSH	0.1–10.0	0.9 (0.7–1.2)	0.538		
B-leucocytes	4.0–11.2	1.0 (1.0–1.1)	0.076		
B-B12 vitamin	147–1475	1.0 (1.0–1.0)	0.157		
B-folate	6.1–54.0	1.0 (1.0–1.0)	0.073		
DAT uptake, putamen	−5.2 to −0.4 SD	0.79 (0.56–1.11)	0.179		
DAT uptake, most affected caudate	−4.7 to 0.1 SD	0.65 (0.50–0.85)	0.002*	0.59 (0.39–0.90)	0.014

Hazard ratio (HR) for the risk of developing dementia (PDD) among 143 patients with Parkinson disease, within 10 years. In the multivariable model, only factors that were significant when corrected for age and sex and that were not excluded because of intercorrelation were retained. *APOE* genotype was investigated by the number of ɛ4 alleles. Eye movement saccades were assessed clinically. Olfactory function was measured by the number of correct answers on the 12-item brief smell inventory test (BSIT).

* The variable was significant after the Benjamini–Hochberg correction for false discovery rate, with *α* = 0.05. MCI, mild cognitive impairment; B, measured in blood; SDs, standard deviations.

**Table 4:** Predictors of cognitive decline in atypical parkinsonism within 10 years

Variable, at baseline	Clinical model	Univariate		Multivariable, adjusted for age and sex	
	Observed range	HR (95% CI)	*P*-value	HR (95% CI)	*P*-value
Age	46.1–87.6 years	1.02 (0.94–1.10)	0.724		
Sex	Male versus female	1.4 (0.5–3.8)	0.552		
Hoehn and Yahr stage	1.5–5.0	1.0 (1.0–1.1)	0.569		
Slow saccades	Yes versus no	3.3 (1.1–9.8)	0.032		
Olfactory function	2–12 correct	0.7 (0.5–0.9)	0.005*	0.68 (0.51–0.91)	0.009
MCI	Yes versus no	0.8 (0.3–2.2)	0.642		
Orthostatic blood pressure drop	–27 to 61 mmHg	1.0 (1.0–1.0)	0.974		
**Biomarkers, at baseline**	**Biomarker model**				
CSF NfL to Aβ_42_ ratio	0.7–10.6	1.1 (0.9–1.4)	0.294		
B-leucocytes	3.5–9.9	1.38 (1.01–1.89)	0.046	1.49 (1.05–2.14)	0.026
DAT uptake, most affected putamen	−5.8 to −2.1 SD	0.5 (0.2–1.1)	0.070		
DAT uptake, most affected caudate	−6.0 to −0.9 SD	0.6 (0.3–1.1)	0.082		

Hazard ratio (HR) for the risk of developing cognitive decline (incident MCI or dementia) during the 10-year follow-up, among 31 patients with atypical parkinsonism (MSA or PSP). Eye movement saccades were assessed clinically. Olfactory function was measured by the number of correct answers on the 12-item brief smell inventory test (BSIT). In the multivariable model, adjustment was made for age and sex.

* The variable was significant after the Benjamini–Hochberg correction for false discovery rate, with *α* = 0.05. B, measured in blood; MCI, mild cognitive impairment.

## Materials and methods

### Study populations

All patients participated in a population-based incidence study of unselected cases of new-onset idiopathic parkinsonism from a geographic catchment area of ≈142 000 inhabitants in northern Sweden (the New Parkinsonism in Umeå study). A population screening procedure was performed to make case identification as complete as possible and to avoid selection bias.^26^ Patients were included in the early motor (drug-naive) phase, between 1 January 2004 and 30 April 2009. After exclusion of patients with secondary parkinsonism (e.g. due to neuroleptic drugs or stroke) or dementia at baseline (e.g. patients with dementia with Lewy bodies), 182 patients were included and followed prospectively. All were assessed by clinical examinations, including the modified Hoehn and Yahr (H&Y), Unified Parkinson’s Disease Rating Scale (UPDRS), Mini-Mental State Examination and oculomotor and autonomic examination, at least yearly. At the latest follow-up, 143 patients were diagnosed with Parkinson disease, 13 with MSA (all with the MSA-Parkinsonism phenotype), 18 with PSP (most with the clinical phenotype of Richardson syndrome), four as having unclassifiable parkinsonism and four did not have idiopathic parkinsonism. A diagnosis of Parkinson disease, MSA or PSP required agreement among the examiners (neurologists specialized in movement disorders) that the clinical criteria for the diagnosis were fulfilled based on the UK Parkinson’s Disease Society Brain Bank criteria^27^ or consensus criteria for MSA or PSP.^28,29^ The diagnosis was neuropathologically verified at autopsy in 10 cases of Parkinson disease and three cases of PSP.

For comparison, 36 healthy control (HC) participants that were age- and sex-matched to the first 50 patients enrolled in the study were included. The controls were recruited by advertisements in the local newspaper, and in a few cases among friends and family of the Parkinson disease participants. Requirements for controls were that they had no neurological disorders, had a normal neurological exam and normal ^123^I-FP-CIT single-photon emission computed tomography (SPECT) brain imaging. All participants provided informed consent. The study was approved by the Regional Medical Ethics Board in Umeå, Sweden, and was performed in accordance with the Declaration of Helsinki.

### Clinical evaluation

For prediction of outcomes, patients were assessed at baseline, in the early disease phase, before the start of dopaminergic medication. Motor function was assessed with the H&Y scale and the UPDRS, with UPDRS subscores divided into tremor score (sum of Items 20 and 21) and postural instability and gait difficulty (PIGD score; the sum of Items 13–15, 29 and 30).^30^ Occulomotor function was investigated clinically by the assessment of blink frequency, saccadic eye movements or findings of ophthalmoplegia. Olfaction was investigated by the 12-item Brief Smell Identification Test,^31^ and depression by the Montgomery Åsberg Depression Rating Scale.^32^ Smoking and alcohol habits and body mass index (BMI) were recorded, and orthostatic blood pressure drop was measured as the systolic pressure difference between the supine position and after standing upright for 3 min. The occurrence of postural instability was assessed throughout the first year. All patients were followed until death or 10 years. During this period, data for mortality and cognitive function (normal, MCI or dementia) were complete. The control participants were followed until 8 years. The patients were assessed in the OFF phase at baseline. At all other time points, patients were assessed in the ON phase for all tests.

### Brain imaging and laboratory and genetic analysis

At study entry, 99 of the patients with Parkinson disease, 21 of the patients with atypical parkinsonism and 30 of the HC participants agreed to CSF collection by standard lumbar puncture in the decubitus position. CSF β-amyloid 1–42 (Aβ_42_) concentration was measured using a sandwich enzyme-linked immunosorbent assay (ELISA) [INNOTEST β AMYLOID (1–42); Fujirebio] constructed to measure Aβ containing the 1st and 42nd amino acids. CSF total tau concentration was measured using a sandwich ELISA (INNOTEST hTAU Ag; Fujirebio, Ghent, Belgium) constructed to determine all tau isoforms irrespective of phosphorylation status. CSF concentrations of tau phosphorylated at threonine 181, α-synuclein and neurofilament light chain protein (NfL) were measured with sandwich ELISAs, as described by the respective manufacturers [INNOTEST PHOSPHO-TAU (181P); Fujirebio, and KHB0061; Invitrogen, Carlsbad, CA, USA and NF-Light; UmanDiagnostics AB, Umeå, Sweden]. The coefficient of variation was 14.0%. All analyses were performed by experienced, board-certified laboratory technicians blinded to the clinical data, using procedures approved by the Swedish Board for Accreditation and Conformity Assessment.

Among the patients with Parkinson disease, 133 agreed to DNA analysis by blood sampling. DNA was isolated from peripheral blood using standard procedures. The variations of interest (the ɛ2/ɛ3/ɛ4 polymorphisms) in the *APOE* gene and the *MAPT* haplotype were genotyped using TaqMan Assays-by-Design (Applied Biosystems, Foster City, CA, USA). The assays were performed according to the manufacturer’s instructions and analysed using the allelic discrimination function of the TaqMan 7900 HT Fast Real-Time PCR system (Applied Biosystems). Peripheral blood measurements of TSH, B12, folate concentrations and leucocyte count were made by standard clinical chemistry tests.

Of the 182 patients enrolled in the study, 170 patients (93.4%) underwent presynaptic dopamine active transporter (DAT) brain imaging by 123I-FP-CIT (DaTSCAN; GE Healthcare BV) SPECT. The imaging protocol was done within the framework of a non-profit clinical trial (EU no. 2009-011748-20) and is further described in Supplementary Methods 1. In brief, normal reference values were derived from 30 age-matched, neurologically HCs and reduction of DAT activity in the patients was measured in standard deviations (SDs) of the normal values. All patients who participated in the DAT imaging in this study (*n* = 163) had a pathologic scan.

### Assessments of cognitive impairment and dementia

Full neuropsychological testing for diagnoses of MCI and dementia was performed at baseline, 1, 3, 5 and 8 years. The tests battery that was used and the criteria for MCI and PDD diagnoses are described in Supplementary Table 1. The MCI diagnoses were made based on Movement Disorder Society Level 2 criteria.^33^ Test performance in specific domains was investigated over time by merging test results into six domains (working memory, attention, visuospatial function, language, episodic memory and executive function), following the partition suggested by the Movement Disorder Society. However, trail making test (TMT) A was used as a measurement of both psychomotor speed and attention, because this domain can be hypothesized to be modifiable by dopaminergic medication. Structural brain MRI and routine laboratory tests were performed to exclude other causes of dementia than Parkinson disease, MSA or PSP. Diagnoses of dementia were determined in accordance with Movement Disorder Society criteria for dementia in Parkinson disease^34^ and, in cases of dementia in PSP, also fulfilling DSM IV criteria for dementia (‘dementia due to other medical conditions’). No patient with Parkinson disease had onset of severe cognitive symptoms 1 year or earlier after motor onset (e.g. as in Lewy body dementia).

### Statistical analysis

Potential demographic or clinical group differences at baseline were examined by one-way analysis of variance, Fisher exact test and Kruskal–Wallis test, and correlation between variables by Spearman *ρ*, as appropriate. Cognitive test scores are presented in *z*-scores (i.e. SD from the mean of the HCs at baseline), and thereby shown in the same scale across domains. Domain scores consist of the mean of all *z*-scores included in each cognitive domain. Longitudinal differences in performance in the cognitive domains (shown in Table 2) are estimated marginal means by linear mixed-effects models, after adjustment for age and gender. The use of mixed models accounts for variability in length of follow-up and is flexible with missing data. Residuals were examined to ensure linear assumptions were fulfilled, and in cases of a non-normal distribution variables were log-transformed to obtain normal distribution before use in the final model. The Kaplan–Meier method is used to estimate the cumulative probability of cognitive decline over time, and the Cox proportional hazards statistic to estimate the influence of candidate risk factors on the time to reach the end-point. The investigated endpoint in Parkinson disease was PDD. Because there were no cases of dementia in multiple system atrophy, any cognitive decline (a decline from normal to MCI or from MCI to dementia) was the investigated endpoint in atypical parkinsonism. A list of potential risk factors for the cognitive decline was generated, including factors previously found predictive, factors possibly differently associated with Parkinson disease and atypical parkinsonism, factors that are potentially modifiable and factors of neurobiological interest (biomarkers). Optimal cut-offs were defined by the highest Youden Index (sensitivity + specificity − 1). Multivariable models were developed, with factors included and retained based on Akaike information criterion. To avoid multicollinearity and overfitting in the final models, all significant factors were investigated for intercorrelation. In cases of intercorrelation, the weakest factor in the model was excluded. For clinical utility, log regression coefficients were calculated for the three most predictive clinical variables and biomarker (CSF Aβ42) for prediction of PDD. Individual risk is calculated by: exp(patient’s risk score)/[1 + exp(patient’s risk score)], where a patient’s risk score = intercept + (*b* Variable 1 × Variable 1) + (*b* Variable 2 × Variable 2) + (*b* Variable 3 × Variable 3) etc., and *b* = regression coefficient. *P*-values of <0.05 were considered significant. However, for univariate comparisons with several tests (all tables except Table 1), we controlled for a false discovery rate of 0.05 by the Benjamini–Hochberg procedure. All statistical analyses were performed using SPSS 23.0 (IBM Corp., Armonk, NY, USA).

### Data availability

Anonymized data can be obtained by request from any qualified investigator for purposes of replicating procedures and results.

## Results

One hundred and seventy-four patients with new-onset, drug-naive, idiopathic parkinsonism (Parkinson disease, MSA or PSP) and 36 neurologically HCs were included in the study and followed up with the complete neuropsychological test battery at 523 different occasions. Parts of the test battery were performed on several additional occasions. At baseline, there were no large demographic differences between groups, but differences in patient characteristics such as cognitive functioning, depression and CSF NfL concentrations (Table 1). All three disease groups showed higher incidences of cognitive decline than in the HCs (Fig. 1A).

### Cognitive impairment in Parkinson disease

At baseline, 43% of the patients with Parkinson disease (62 of 143 patients) had MCI, which was 32% more than in the HCs (Table 1). Patients with Parkinson disease also had a higher incidence of any cognitive decline (Fig. 1A). By 5 years, 39 cases of PDD had occurred, and the cumulative probability of PDD was 30%. By 10 years (at the end of the study), 64 cases of PDD had occurred, and the cumulative probability of PDD was 54% (Fig. 1B). The incidence rate of dementia was 56.2 cases [95% confidence interval (CI) 44.2–71.2] per 1000 patient years. At 10 years, the mean survival was 8 years for all patients with Parkinson disease. The proportion of patients alive without dementia was 28% (40 patients) at 10 years, and the proportion alive without any evidence of cognitive impairment was 18% (26 patients). The cumulative probability of any cognitive decline was 74%. At death or the end of the study, 46 patients (32% of all patients with Parkinson disease) had experienced MCI without dementia (i.e. stable MCI), during an average period of 4.4 years. The patients with stable MCI (operationally defined as MCI for 2 years or more) had better olfactory function and higher Aβ42 in CSF compared with the other patients (data not shown) but were otherwise similar.

Compared with the HCs, patients with Parkinson disease had lower scores in all tested cognitive domains (both by difference at baseline and overall test occasions, Table 2). The largest impairments occurred in psychomotor speed and attention, as measured by TMT A (2.19 SD slower performance, *P* < 0.001), episodic memory (0.87 SD lower scores, *P* < 0.001) and executive functioning (1.13 SD lower scores, *P* < 0.001) (Table 2B). The psychomotor speed and attention improved by 9% (*P* = 0.046) between baseline and Year 1, following dopaminergic treatment.

Baseline Parkinson disease traits that predicted future dementia in univariate analyses, after applying the Benjamini–Hochberg procedure to correct for false discovery rate, were, in the order from stronger to weaker factors: MCI, poorer olfactory function, overall disease severity (as measured by UPDRS III subscores, total UPDRS and H&Y stage), age, PIGD, lower blink frequency, symmetrical motor onset, no alcohol consumption, blood pressure drop, lower level of education and occurrence of postural instability during the first year (Table 3 and Fig. 3). Of these, education, H&Y stage, alcohol use and blood pressure drop were not significant predictors after adjustment for age and sex. Among the biomarkers, high NfL and low Aβ42 in CSF, low DAT activity in the most affected caudate nucleus, and, after adjustment for age and sex, higher BMI, predicted PDD. Standard blood work was not predictive.

Baseline clinical factors that were independently related to the risk of PDD were included in a multivariable model (Table 3). After excluding intercorrelated factors, a clinical model including MCI, UPDRS III, olfactory function and age showed the highest accuracy for the prediction of PDD. Of these four factors, age had the weakest association (Table 3) and was excluded in the logistic regression model that was used to calculate individual risk (Fig. 4C) to avoid overfitting. Biomarkers predictive of future dementia in Parkinson disease were the NfL to Aβ42 ratio and DAT deficit in the caudate. For clinical usefulness, the strongest predictors were retained, rendering a simplified model with Aβ42 in CSF added to the clinical model (Fig. 4). The addition of CSF Aβ42 significantly improved the accuracy in predicting dementia, improving the area under the curve (AUC) from 0.77 to 0.86 (both *P* < 0.001). The regression coefficients from this combined model can be used to quantify individual risk. The model shows a highly variable but also predictable dementia risk in Parkinson disease (Fig. 4); e.g. as demonstrated by a 10-year risk of 9% in a patient with Aβ42 in the highest tertile and normal cognition compared with a 85% risk in a patient with Aβ42 in the lowest tertile and MCI.

### Cognitive impairment in MSA

At baseline, 23% (3 of 13 patients) of the patients with MSA had MCI; 11% more than in the HCs (Table 1). Patients with MSA also had a higher incidence of MCI over time compared with the HCs (Fig. 1A, *P* = 0.016, log rank). No patient with MSA developed dementia during follow-up. The mean survival was 5 years. The cumulative probability of any cognitive decline prior to death or end of the study was 36% (Fig. 3). At death or the end of the study, six patients (46% of all patients with MSA) had experienced stable MCI, during an average period of 3 years. Compared with the HCs, the patients with MSA had lower scores for psychomotor speed, episodic memory and executive function over time (Table 2 and Fig. 2). Patients with MSA were not different compared with Parkinson disease (Table 2 and Fig. 2), with the exception that the psychomotor speed and attention did not improve between baseline and 1 year, following dopaminergic treatment.

### Cognitive impairment in PSP

At baseline, 77% (14 of 18 patients) with PSP had MCI; a 66% higher prevalence than in the HCs. Patients with PSP had a higher incidence of cognitive decline compared with the HCs (Fig. 1A, *P* < 0.001, log rank). The mean survival was 5.4 years. By 5 years, eight cases of dementia had occurred, and the cumulative probability of dementia was 51%. By 10 years, 10 cases of dementia had occurred, and the cumulative probability of dementia was 71% (Fig. 1B). The incidence rate of dementia in PSP was 103.1 cases (95% CI 57.3–185.2) per 1000 patient years. The cumulative probability of any cognitive impairment prior to death or end of the study was 79%. At death or the end of the study, seven patients (39% of all patients with PSP and including the two surviving patients) had experienced stable MCI without dementia, during an average period of 5.4 years. MCI or dementia affected nearly all with PSP during follow-up (17 of 18 patients). Compared with the HCs, the patients with PSP had lower scores in all tested cognitive domains (Table 2). The type of cognitive dysfunction differed from the cognitive dysfunction in Parkinson disease, with poorer performance in language, episodic memory and psychomotor speed and attention as measured by TMT A (Table 2 and Fig. 2). Similar to MSA, the psychomotor speed and attention did not improve between baseline and 1 year following dopaminergic treatment.

### Predictors of cognitive decline in atypical parkinsonism

Baseline predictors of cognitive decline in idiopathic, atypical parkinsonism were impaired olfaction, slow eye movement saccades and peripheral inflammation as measured by increased plasma leucocytes (Table 4). These factors were all significant after adjustment for age and sex. The associations were mostly driven by the patients with PSP. Striatal DAT activity in atypical parkinsonism showed a trend for similar, negative relationship with cognitive impairment as in Parkinson disease which was however not significant. If applying stringent correction for false discovery rate (*α* = 0.05) only impaired olfaction predicted cognitive decline.

### Intercorrelation of predictive factors

Higher age correlated with UPDRS III score (rs = −0.40, *P* < 0.001), olfaction (rs = −0.28, *P* < 0.001), blood pressure drop (rs = 0.31, *P* < 0.001), leucocyte count (rs = 0.33, *P* < 0.001), level of education (rs = −0.58, *P* < 0.001) and performance in all tested cognitive domains (rs between −0.20 and −0.49, *P* < 0.005) in Parkinson disease. In atypical parkinsonism, age correlated only with olfaction (rs = −0.42, *P* = 0.040) and performance in some cognitive domains (rs between −0.40 and −0.49, *P* < 0.05), but not with working memory, visuospatial function or episodic memory. In Parkinson disease, the NfL concentration in CSF correlated with UPDRS III scores (rs = 0.36, *P* < 0.001), lower DAT activity in the caudate (rs = −0.21, *P* = 0.037, poorer cognitive performance in psychomotor speed, language, episodic memory and executive function (rs between −0.30 and 0.39, *P* < 0.005) and with age (rs = 0.63, *P* < 0.001). Other correlations with NfL are described in a previous publication.^18,35^ The CSF Aβ42 concentration did not correlate with other factors in Parkinson disease, with the exception that carriers of a higher number of *APOE* ɛ4 alleles had lower Aβ42, rs = −0.23, *P* = 0.026. Lower DAT activity in the caudate nucleus in Parkinson disease correlated with UPDRS III scores (rs = 0.31, *P* < 0.001), olfaction (rs = −0.23, *P* = 0.009) and NfL in CSF.

Considering potentially modifiable factors, patients with Parkinson disease who did not consume any alcohol were older (rs = 0.28, *P* < 0.001), had lower level of education (rs = −0.19, *P* = 0.042), higher UPDRS III scores (rs = 0.35, *P* < 0.001) and poorer cognitive test scores. BMI was unrelated with other investigated factors. Because higher blood leucocyte count predicted cognitive impairment in atypical parkinsonism, it was also investigated in Parkinson disease. Two patients with Parkinson disease with high leucocytes due to infection or blood disease were excluded prior to this analysis. In Parkinson disease, increased leucocytes correlated with higher UPDRS III scores (rs = 0.19, *P* = 0.027), lower DAT activity in the caudate (rs = −0.20, *P* = 0.025), poorer cognitive performance in psychomotor speed, language, episodic memory and executive function (rs between −0.25 and −0.44, *P* < 0.01), and higher NfL in CSF, but all factors, except performance in language and executive function, were unrelated with leucocytes after age adjustment. In atypical parkinsonism, a higher leucocyte count did not correlate with age but showed negative correlations with performances in psychomotor speed (rs = −0.32, *P* = 0.002), episodic memory (rs = −0.64, *P* < 0.001) and the Aβ42 concentration in CSF (rs = −0.51, *P* = 0.031). These correlations remained significant after age adjustment.

## Discussion

To our knowledge, this prospective study represents the first detailed, longitudinal comparison of different cognitive functions in new-onset Parkinson disease and atypical parkinsonism, in a population-based setting. The results of the study, which involved detailed in-person examination of all participants, show that the rate of cognitive decline and dementia is markedly different in the three most common idiopathic parkinsonian diseases. The accumulated risk of dementia over 10 years (the cumulative probability) was 54% in Parkinson disease and 71% in PSP, while no cases of dementia occurred in MSA (or in the neurologically HCs). The incidence rate of dementia in PSP was almost double the rate found in Parkinson disease, with 103.1 versus 56.2 cases per 1000 lived patient years.

The cumulative probability of dementia in Parkinson disease, of 54%, is similar to estimates in the few other population-based cohort studies of dementia in incident Parkinson disease that have been reported to date.^5,36^ Taken together, these data confirm that about half the population with Parkinson disease will develop dementia in Parkinson disease within 10 years from the first visit, while considering that the death rate is higher in these patients.^3^ The slightly higher figure in the present study compared with the study by Williams-Gray *et al*.^5^ could be explained by higher ages at baseline in the present study. However, the incidence of dementia is lower than figures from other studies that are often cited.^4^ Notably, about half of all patients with Parkinson disease did not develop dementia during the 10-year follow-up period.

### Cognitive phenotypes in the different parkinsonian diseases

Patients with PSP had a marked deterioration in the language (including verbal fluency), episodic memory and psychomotor speed and attention, showing more impairments in these domains than in Parkinson disease and markedly more than in HCs. This cognitive phenotype overlaps with dementia phenotypes seen both in Lewy body dementia, Alzheimer disease and PDD.^37,38^ and is consistent with previous studies of PSP.^1,39–41^ These results indicate that the tauopathy of PSP affects brain networks of importance for cognitive functions more aggressively and more rapidly than the α-synucleinopathy of Parkinson disease and MSA. The marked deterioration in language, episodic memory and psychomotor speed and attention may be detected clinically, using neuropsychological tests as a tool to distinguish PSP from Parkinson disease and MSA. Furthermore, similar to MSA, psychomotor speed and attention did not improve after dopaminergic treatment in PSP, while such an effect was visible in Parkinson disease.

In MSA, qualitatively similar cognitive impairment compared with Parkinson disease was found, compatible with previous studies.^1,42,43^ In MSA, the presence of significant cognitive decline is an exclusion feature by current consensus criteria.^29,28,43^ Nevertheless, dementia can occur in MSA with estimates reported in the range of 14–16%.^44,45^ The high mortality in MSA and PSP, with a 3.3 times higher age- and sex-standardized mortality compared with a Swedish control population in the present cohort,^3^ makes it difficult to determine the true, long-term risk of dementia in these diseases.

### Prediction of dementia in early Parkinson disease

In Parkinson disease, models to calculate the individual risk of dementia with high precision have value for several purposes. For instance, such models can be used for the selection of patients with high risk into clinical trials, reducing patient heterogeneity, costs and follow-up needed to reach cognitive end-points and may help to predict the response to treatments. This study implements a new, integrated clinical-biomarker model for the prediction of PDD, using longer follow-up than has been achieved for previous models. The clinical risk profile that had the strongest association with PDD development (MCI, hyposmia, motor disease severity and age) shows risk factors that are consistent with previous studies in the same cohort, at earlier time points,^13,14^ and with multiple other studies.^15^ However, studying these factors together as PDD predictors over 10 years enabled an accurate prediction of dementia (AUC: 0.77), and adding biomarkers in CSF to the clinical model improved the predictive accuracy further. The combined clinical-biomarker model shows an excellent accuracy in predicting whether a patient will develop dementia within 10 years (AUC: 0.86, Fig. 4A). It demonstrates that dementia in Parkinson disease is highly variable but predictable. The optimized model can be implemented in any setting with access to olfaction tests and an accredited CSF analysis laboratory.

Notably, most major risk factors of PDD in the model have been found to correlate either with the underlying core pathology of Parkinson disease (i.e. striatal, including caudate DAT deficits and hyposmia are correlated with nigrostriatal degeneration and the spread of α-synuclein) or with Alzheimer disease co-pathology (which is correlated with low Aβ42 in CSF).^46–48^ Our findings therefore point to these general classes of neurodegenerative disease pathologies as main drivers of cognitive impairment in Parkinson disease.

### Biomarkers of cognitive decline

This study reveals a negative association of higher CSF NfL with performance in psychomotor speed, episodic memory, language and executive function in early, new-onset Parkinson disease, and that this finding translates to a higher risk of future PDD. Because NfL is a sensitive marker of axonal degeneration,^49^ the finding indicates that axonal degeneration may be an early mechanism in the chain of events leading to cognitive decline. Indeed, there is evidence that dopaminergic axonal degeneration is an early feature of Parkinson disease.^50,51^ In the present cohort, we previously found that high NfL in early Parkinson disease correlates with reduced striatal DAT activity and with motor disease severity, especially in bradykinesia and gait measures.^35^ The relation between high NfL and cognitive deficits shown here is also compatible with a recent cross-sectional study.^52^ The most accurate biomarker for the prediction of PDD was the combination of NfL with Aβ42 in CSF. In the group of Parkinson disease patients with a lower baseline NfL than Aβ42 (with a ratio <1) in this study, only three patients developed dementia. The performance of this biomarker-based prediction does not seem to deteriorate over 10 years (Fig. 3A) and is more strongly predictive than age, which indicates the identification of pathophysiologically distinct Parkinson disease subtypes. When using all baseline clinical variables and biomarkers to optimize PDD prediction, however, NfL did not improve the model significantly. The explanation for this was that, while CSF Aβ42 is independently predictive, NfL is intercorrelated with other highly predictive factors (such as UPDRS and olfactory function). Therefore, CSF Aβ42 was the only biomarker retained in the final, parsimonious prediction model. In addition, although we found a negative correlation between the number of ApoE ɛ4 alleles and Aβ42 in CSF, and in contrast to several large cohort studies,^53,54^ we did not find a significant genotype effect on dementia risk (here considering ApoE and MAPT genotypes). This could be explained by smaller effect sizes on the risk of dementia by genotype, relative to the other predictive factors investigated in this study. Many other biomarkers (such as GBA mutations or heart fatty acid-binding protein in CSF) could be considered for PDD prediction, but the model presented here is reasonably simple and does not require genetic counselling and is therefore likely to be implementable in a clinical context.

Our findings are not altogether encouraging regarding preventable risk factors for dementia in Parkinson disease. Alcohol consumption, as compared with no alcohol consumption, was associated with a slightly lower risk of PDD (Table 3), which was not significant after age adjustment. This indicates that the higher risk for dementia was largely explained by older age in the group that consumed no alcohol. A risk-increase for PDD was associated with higher BMI, which remained significant after age- and sex-adjustment. It is far from clear that higher BMI causes a higher risk of PDD (an alternative being that BMI is associated with the brain pathology that causes PDD, e.g. by association with inactivity). Even so, it seems reasonable to recommend physical activity to maintain cognitive function and well-being in Parkinson disease, in particular since a protective effect may be supported by other studies.^21,22^

### Predictors of cognitive decline in atypical parkinsonism

Although hyposmia had a significant influence in all three parkinsonian diseases, slow eye movement saccades, as assessed clinically, and increased blood leucocytes predicted cognitive decline specifically in atypical parkinsonism. These findings were most evident in PSP. In PSP, slow eye movement saccades are sensitive indicators of the underlying disease processes, and occulomotor impairments have been shown to be closely related to the impairment of spatial attention and spatial short-term memory.^55,56^ Although a simple clinical examination of eye movements is very likely to miss subtle saccadic slowing, this study indicates that such an examination may nonetheless capture an increased risk of cognitive decline. Recent studies have also found that brain neuroinflammation is associated with the severity of symptoms in PSP.^57,58^ Such neuroinflammation co-localizes anatomically with tau pathology in the brain^59^ and patients with PSP also show increased levels of IL-2 and shifts in peripheral T-cell populations.^60^ Notably, increased leucocytes in this study correlated with lowered CSF Aβ42 levels. A possible explanation for the difference with Parkinson disease in this study is that more aggressive forms of neurodegeneration (as seen in PSP) may trigger a peripheral immune response, and thereby constituting a biomarker of risk of cognitive decline. This could be especially true in the PSP-Richardson syndrome, because our inclusion criteria may have made our study skewed towards this phenotype.

Interestingly, MCI that is stable (not progressing to dementia) for several years seems to be common in idiopathic parkinsonism. Throughout this study, 32% of the patients experienced stable MCI in Parkinson disease, for at least 2 years, without development of dementia throughout the study, compared with 46% of the patients with MSA, and 39% of the patients with PSP. The finding that stable MCI in Parkinson disease is associated with better olfactory function and higher CSF Aβ42 indicates that stable MCI is characteristic for a milder phenotype of Parkinson disease.

### Limitations

Because fewer patients were alive and tested with the full neuropsychological test battery at 5 and 8 years, compared with earlier occasions, the measured cognitive dysfunctions may be an underestimation of the true cognitive dysfunction. This could be especially true in atypical parkinsonism. Even so, the classification of cognitive impairment was complete over 10 years in this population-based cohort. Further strengths were that all patients were investigated in the early, non-medicated phase, were well-characterized by multimodal investigations, and that the predictor model was built and tested prospectively. Weaknesses were that oculomotor and postural instability assessments were performed by clinicians and therefore are likely to be subject to intra- and inter-rater variability. In addition, patients with PSP predominantly had the Richardson syndrome phenotype and there were no cases of MSA-C among the patients with MSA. Few pathologically confirmed diagnoses were available. However, all patients who performed DAT imaging had a pathological scan and the rate of cognitive impairment in those with a confirmed pathological diagnosis was comparable to that of the sample as a whole.

## Conclusions

The three most common idiopathic parkinsonian diseases (Parkinson disease, MSA and PSP) have distinguishing cognitive phenotypes. In the absence of disease-modifying treatment for dementia in Parkinson disease, the use and communication of precise predictions regarding this severe complication require ethical consideration and sensitivity to the preference of the individual patient. Even so, medical advance pushing beyond present practice models is likely to be needed to make progress in the prevention and treatment of PDD.

## Supplementary Material

fcac040_Supplementary_Data

## Abbreviations

Aβ42 (amyloid-β_42_) =: β-amyloid 1–42
DAT =: dopamine active transporter
ELISA =: enzyme-linked immunosorbent assay
H&Y =: Hoehn and Yahr
MCI =: mild cognitive impairment
NfL =: neurofilament light chain protein
PDD =: Parkinson disease dementia
PIGD =: postural instability and gait disorder
SPECT =: single-photon emission computed tomography
TMT =: trail making test
UPDRS =: Unified Parkinson’s Disease Rating Scale
