# Supplementary material for: Prediction and early biomarkers of cognitive decline in Parkinson disease and atypical parkinsonism: a population-based study
Source: Brain Commun. 2022 Mar 15;4(2):fcac040. doi: 10.1093/braincomms/fcac040 (PMC8947320; doi:10.1093/braincomms/fcac040)
Supplement: fcac040_Supplementary_Data [file fcac040_supplementary_data.docx]

**Supplementary material**

1. **Supplementary methods 1**

**Striatal DAT imaging**

Striatal DAT imaging by single-photon emission computed tomography (SPECT) was done 3 hours following an IV bolus dose of 185MBq 123I-FP-CIT. Imaging was done prior to commencement of medication at baseline. Semiquantitative analysis (based on regions of interest) and visual evaluation of the DAT SPECT were done unbiased by clinical information. The imaging protocol, equipment, and semiquantitative evaluation have been described earlier.18 Two different SPECT cameras were used during the course of the project; one brain-dedicated SPECT camera (the Neurocam) was later substituted by a multipurpose hybrid SPECT/CT (both General Electric, Milwaukee, WI).

Normal reference values were established for both equipments (see: Mo SJ, Linder J, Forsgren L, Larsson A, Johansson L, Riklund K. Pre- and postsynaptic dopamine SPECT in the early phase of idiopathic parkinsonism: a population-based study. Eur J Nucl Med Mol Imaging 2010;37:2154–2164, and Jakobson Mo S, Larsson A, Linder J, et al. 12│I-FP-Cit and 123I-IBZM SPECT uptake in a prospective normal material analysed with two different semiquantitative image evaluation tools. Nucl Med Commun 2013;34:978–989).

1. **Supplementary table 1:**
2. **Neuropsychological functions and cognitive tests used for classification of cognitive impairment**

**Neuropsychological function Test**

**Episodic memory:** Free and Cued Selective Reminding Test (FCSRT)^a^

Logical memory and Paired associative learning from Wechsler memory scale (WMS)^a^

Brief visuospatial memory test (BVMT) total recall^a^

**Working memory:**  Digit span forward, from Wechsler Adult Intelligence Scale (WAIS) III^b^

Digit span backwards, from Wechsler Adult Intelligence Scale (WAIS) III^b^

**Psychomotor speed and attention:** Trail Making Test (TMT) A^c^

**Language:** Boston Naming Test (BNT)

Semantic fluency (letters and animals in 60 seconds)

**Visuospatial function:** The Benton Judgement of Line Orientation test^d^

Pentagon copying from Mini Mental State Examination (MMSE)

**Executive function:** Wisconsin card sorting test (WCST) – computer version 2^e^

Mental Control from Wechsler memory scale (WMS)

Shiftcost (TMT B – TMT A)^e^

**A domain score** was calculated by the mean of standardized scores (Z-scores) in the tests of episodic memory^a^, working memory^b^, psychomotor speed and attention^c^, language (all tests), visuospatial function^d^ and executive function^e^.

1. **Subjects were classified as having mild cognitive impairment if:**

• Impaired in a minimum of two tests in one domain (single domain MCI) or in a minimum of one test in two different domains (multiple domain MCI).

• Impairments were ≥1.5 standard deviations below mean of normative data.

• Self-perceived cognitive decline was reported in the Parkinson’s Disease Questionnaire-39 (PDQ-39) and/or directly by the patient and/or cognitive decline was reported by family member.

• There was no functional impairment in basic activities of living (i.e. driving a car, social or personal care, medication management).

1. **Dementia diagnosis:**

In addition to the extensive neuropsychological testing at 0, 1, 3, 5 and 8 years, incident dementia in Parkinson disease (PDD) was diagnosed on the basis of previous neuropsychological test results, Mini Mental State Examination (MMSE) decline, cognitive decline reported by patient and/or family member and by the occurrence of functional impairment in basic activities of living due to cognitive decline. Dementia diagnosis was established by neurologists specialized in movement disorders, experienced with age-dependent neurodegenerative disorders including dementia. A diagnosis of dementia in Parkinson disease was established when clinical criteria were fullfilled according to MDS PDD criteria (Emre M, Aarsland D, Brown R, et al. Clinical diagnostic criteria for dementia associated with Parkinson's disease. *Mov Disord*. Sep 2007;22(12):1689-707; quiz 1837. doi:10.1002/mds.21507), including:

- A diagnosis of Parkinson disease
- Impairment in more than one cognitive domain
- A decline from premorbid level
- Deficits severe enough to impair daily life (social, occupational, or personal care), independent of the impairment ascribable to motor or autonomic symptoms
- Associated clinical behavioural features and lack of other explanations for cognitive dysfunction such as drug intoxication or major depression.

To allow comparison of dementia across all three diseases, the criteria for a diagnosis of dementia in MSA and PSP were the same as for dementia in Parkinson disease (PDD). However, because these PDD criteria are not constructed to be formal criteria for diagnoses of dementia in MSA and PSP, the diagnosis of dementia in PSP or MSA was also determined to fullfill the dementia criteria according to DSM (DSM IV “dementia due to other medical conditions”). Applying these additional dementia criteria did not change any of the diagnoses of dementia established in this study.
